# Combined Fish Oil and Pine Bark Extract Supplementation Improves Short-Term Memory and the Antioxidant Status in Middle-Aged and Older Adults with Mild Cognitive Impairment: A Randomized Double-Blind, Parallel-Group Pilot Study

**DOI:** 10.3390/antiox15050588

**Published:** 2026-05-06

**Authors:** Tse-Chia Hsiao, Cheng-Dien Hsu, Qian Xiao, Yi-Hsiu Chen, Yannick Piriou, Hitoshi Shirakawa, Suh-Ching Yang

**Affiliations:** 1 School of Nutrition and Health Sciences, College of Nutrition, Taipei Medical University, Taipei 11031, Taiwan; jamie.hsiao@nenc.com.tw (T.-C.H.); ma07106012@tmu.edu.tw (Q.X.);; 2 Department of Psychiatry, Taiwan Adventist Hospital, Taipei 10556, Taiwan; alexhsu@tahsda.org.tw; 3 QWB, Novapole, Zone de la Nau, 2 rue des Fresnes, 19240 Saint-Viance, France; yacons@outlook.fr; 4 Laboratory of Nutrition, Graduate School of Agricultural Science, Tohoku University, Sendai 980-8857, Japan; shirakah@m.tohoku.ac.jp; 5 Research Center of Geriatric Nutrition, College of Nutrition, Taipei Medical University, Taipei 11031, Taiwan; 6 Nutrition Research Center, Taipei Medical University Hospital, Taipei 11031, Taiwan; 7 School of Gerontology and Long-Term Care, College of Nursing, Taipei Medical University, Taipei 11031, Taiwan

**Keywords:** mild cognitive impairment, fish oil, pine bark extract, cognitive function, antioxidative status

## Abstract

Mild cognitive impairment (MCI) represents an intermediate stage between normal cognitive function and dementia. Delaying the onset or progression of dementia has therefore become a key research priority. Although previous studies have examined the individual effects of fish oil or pine bark extract on cognitive decline, their findings remain inconclusive. In this study, we compared the effects of fish oil alone versus fish oil combined with pine bark extract on cognitive function and the oxidative status in patients with MCI. Participants aged 55–75 years with MCI were enrolled in a 24-week, double-blind, parallel-group trial, and they were randomly assigned to either a fish oil group (F group, *n* = 14), which received one fish oil capsule (350 mg eicosapentaenoic acid and 250 mg docosahexaenoic acid) and one placebo capsule, or a fish oil combined with pine bark extract group (F+P group, *n* = 14), which received one fish oil capsule and one pine bark extract capsule (100 mg). Compared to the baseline, the F group showed a significant decrease in Clinical Dementia Rating scores and a significant increase in Mini-Mental State Examination scores. In the subdomain analysis of the Cognitive Abilities Screening Instrument, the F group demonstrated a significant increase in the drawing score, whereas the F+P group showed a significant increase in the short-term memory score. Regarding the antioxidant status, compared to the baseline, the F group exhibited a significant increase in plasma thiobarbituric acid reactive substance (TBARS) levels and erythrocytic superoxide dismutase activity, whereas catalase (CAT) activity significantly decreased. After 24 weeks, plasma TBARS levels showed no significant change, while CAT activity was significantly higher in the F+P group than in the F group. These findings suggest that combined supplementation with fish oil and pine bark extract may be associated with potential improvements in short-term memory performance and antioxidant status in middle-aged and older adults with MCI, although the results should be interpreted cautiously.

## 1. Introduction

Dementia is a major global health concern and a leading cause of disability and dependency among older adults worldwide [1]. It is estimated that more than 55 million people are currently living with dementia globally, and this number is projected to reach 78 million by 2030 and 139 million by 2050 [2,3]. As population aging accelerates, identifying modifiable risk factors and preventive strategies for cognitive decline has become increasingly important.

Mild cognitive impairment (MCI) represents an intermediate stage between normal cognitive function and dementia [4], and individuals with MCI have a significantly higher risk of progressing to dementia than cognitively normal individuals [5]. Because early cognitive changes are often subtle and overlooked, it has been estimated that 27–65% of individuals with MCI do not receive timely interventions and have an average progression time of approximately 3–5 years [5,6,7,8,9]. Lifestyle-related factors, including suboptimal dietary patterns, smoking, alcohol use, obesity, and physical inactivity, have been identified as modifiable risk factors for cognitive decline [10]. Evidence suggests that lifestyle interventions at the MCI stage, including dietary modifications and nutritional supplementation, may delay progression and potentially restore cognitive function [11]. Accordingly, World Health Organization guidelines recommend dietary and nutritional improvements as part of lifestyle strategies to reduce the risk of progression from MCI to dementia [10].

Oxidative stress and chronic inflammation are considered central mechanisms underlying neurodegeneration and cognitive decline. Increased reactive oxygen species (ROS) production, impaired antioxidant defense, and lipid peroxidation have been implicated in neuronal damage and the progression of cognitive impairment [12,13]. Nutritional factors with antioxidants and anti-inflammatory properties have therefore received considerable attention for their potential role in maintaining cognitive function. Among these, fish oil, which is rich in n-3 polyunsaturated fatty acids (FAs and PUFAs), particularly eicosapentaenoic acid (EPA) and docosahexaenoic acid (DHA), has been widely studied for its anti-inflammatory, neuroprotective, and membrane-stabilizing properties [14]. Epidemiological studies suggest that higher fish consumption or circulating n-3 PUFA levels are associated with reduced risks of cognitive decline and dementia [15,16]. However, supplementation with fish oil rich in n-3 PUFAs may also increase susceptibility to lipid peroxidation due to their high degree of unsaturation, highlighting the importance of the antioxidant balance [17,18]. Previous clinical and epidemiological studies have yielded inconclusive findings regarding the effects of n-3 PUFAs on dementia risk and progression, suggesting that additional factors, such as the oxidative balance and antioxidant status, may influence the efficacy of n-3 PUFA supplementation [19].

Pine bark extract, particularly standardized maritime pine bark (*Pinus pinaster* Aiton) extract rich in oligomeric proanthocyanidins (typically 65–75%) and other phenolic compounds, has been shown to possess strong antioxidant capacities, attenuate lipid peroxidation, and improve endothelial function in both experimental and clinical settings [20,21,22,23]. In addition to its antioxidant effects, pine bark extract was reported to exert anti-inflammatory and neuroprotective actions, potentially through modulating oxidative stress-related signaling pathways and improving cerebral microcirculation [24]. Some clinical studies suggest that supplementation with pine bark extract may improve cognitive performance, attention, and/or mental fatigue in older adults and individuals with MCI, although findings remain limited and heterogeneous [23,25]. Moreover, in our previous study, 4-week supplementation with 25 or 50 mg of pine bark extract significantly ameliorated inattention and impulsivity, increased the erythrocytic reduced glutathione (GSH)/glutathione disulfide (GSSG) ratio, and decreased plasma thiobarbituric acid reactive substance (TBARS) levels in children with attention deficit hyperactivity disorder (ADHD) [26].

Because n-3 PUFAs are highly susceptible to lipid peroxidation despite their anti-inflammatory and neuroprotective properties, co-supplementation with polyphenol-rich antioxidants may help maintain the redox balance and enhance the functional benefits of n-3 PUFAs. Such a combined strategy may provide synergistic protection against oxidative stress-related cognitive decline; however, clinical evidence on the joint effects of fish oil and polyphenol-rich antioxidants on oxidative stress and antioxidant defense remains limited. Therefore, in the present study, we investigated the effects of fish oil supplementation with and without pine bark extract on cognitive function and the antioxidative status in individuals with MCI.

## 2. Materials and Methods

### 2.1. Study Design and Participant Recruitment

This study was approved by the Joint Institutional Review Board of Taipei Medical University (approval no. N202012034; approval date: 2 February 2021). The trial was also registered at ClinicalTrials.gov (identifier: NCT05573269; registration date: 25 September 2022). Participant recruitment began on 11 November 2021, and all post-intervention data collection was completed by 12 August 2023.

Recruitment was conducted in the psychosomatic medicine and psychiatry outpatient clinics of Taiwan Adventist Hospital and Jingmei Hospital. Participants aged 55–75 years with a diagnosis of MCI were enrolled. In this study, MCI was defined as a Clinical Dementia Rating (CDR) of ≤0.5 and a Mini-Mental State Examination (MMSE) score of 26–30. The Geriatric Depression Scale (GDS) was administered to screen for and exclude participants with depressive symptoms or other emotional conditions that might affect cognitive function. All cognitive and psychological assessments were conducted by a clinical psychologist, and the final diagnosis was confirmed by a psychiatrist. Participants were excluded based on the following criteria: (1) the presence of chronic diseases, including hypertension, diabetes mellitus, hyperlipidemia, cardiovascular disease, liver disease, kidney disease, gastrointestinal disease, or cancer; (2) current use of specific medications, including antihypertensive agents or anticoagulants; (3) an inability to comply with the scheduled intake of the study capsule supplements; and (4) an inability to attend follow-up visits for anthropometric measurements, blood sampling, or psychological assessments.

### 2.2. Intervention

This study was a prospective, double-blind, randomized, parallel-group trial. Randomization was performed by drawing lots, and participants were assigned to one of two groups: a fish oil plus placebo group (F group) and a fish oil combined with pine bark extract group (F+P group). The intervention period lasted 24 weeks. Participants in the F group received one capsule of fish oil and one placebo capsule daily. The dosage of fish oil was determined based on previous studies by Yurko-Mauro et al. [27] and Hashimoto et al. [28]. Each fish oil capsule (VIVA Omega-3™, Viva Life Science, Costa Mesa, CA, USA) contained 350 mg EPA, 250 mg DHA, and 3.5 mg α-tocopherol. Participants in the F+P group received one capsule of fish oil and one capsule of pine bark extract daily. Each capsule contained 100 mg of pine bark extract (a commercial *Pinus pinaster* Aiton, French maritime pine bark extract supplied by Formosa Produce Company, New Taipei City, Taiwan), comprising 65–75% oligomeric proanthocyanidins and complying with the quality specifications of the United States Pharmacopeia (USP 42). The dosage was determined based on previous studies by Belcaro et al. [23] and Ryan et al. [25]. Participants, investigators, and outcome assessors were blinded to the intervention assignments. The active supplement and placebo were prepared in identical opaque capsules and packaged in coded containers to ensure blinding.

Participants received a 12-week supply of the study supplements at weeks 0 and 12. Monthly telephone follow-ups were conducted by research staff to monitor participants’ health status and adherence to the study protocol. Anthropometric measurements, blood pressure (BP), 24 h dietary recall, blood samples, and cognitive function were assessed at weeks 0 (the baseline) and 24.

### 2.3. Anthropometric and Blood Pressure (BP) Measurements

Anthropometric measurements included body weight, body mass index (BMI), body fat mass, and muscle mass, which were assessed using a body composition analyzer (HBF-371, TANITA, Tokyo, Japan). Systolic BP (SBP) and diastolic BP (DBP) were assessed with an automated blood pressure monitor (JPN600, OMRON Healthcare, Kyoto, Japan).

### 2.4. Cognitive Function Assessments

#### 2.4.1. Clinical Dementia Rating (CDR)

The CDR scale was administered by a clinical psychologist to assess the severity of cognitive impairment across six domains: memory, orientation, judgment and problem-solving, community affairs, home and hobbies, and personal care. Each domain is rated from 0 (none) to 3 (severe), with 0.5 indicating questionable impairment; higher scores reflect greater severity. In individuals with MCI, CDR scores are typically 0 or 0.5.

#### 2.4.2. Mini-Mental State Examination (MMSE)

The MMSE is a 30-point screening tool for global cognitive function that assesses orientation, attention, memory, language, and visuospatial ability [29]. Higher scores indicate a better cognitive performance. Education-adjusted criteria were applied: participants with no formal education were required to have an MMSE score of >17, and those with formal education were required to have a score of >24 to be considered within the normal cognitive range. In contrast, eligibility for pharmacological treatment in dementia typically requires MMSE scores of 10–26 together with a clinical diagnosis of dementia.

#### 2.4.3. Cognitive Abilities Screening Instrument (CASI)

The CASI is a multidomain cognitive test with a total score of 100. Compared to the MMSE, the CASI evaluates a broader range of cognitive functions and is more sensitive to early cognitive changes. The CASI evaluates long-term memory (10 points), short-term memory (12 points), attention (8 points), concentration or mental manipulation (10 points), orientation (18 points), language (10 points), drawing or visuospatial construction (10 points), abstract thinking and judgment (12 points), and verbal fluency (10 points). Higher scores indicate a better cognitive performance. The Chinese version (CASI C-2.0) was used in this study. For individuals with ≥6 years of education, a CASI score of 80–100 is generally considered within the normal to MCI range [30,31].

#### 2.4.4. The Geriatric Depression Scale (GDS)

Depressive symptoms were assessed using the GDS, a widely used self-reported screening tool for depression in older adults. The GDS consists of a series of yes/no questions designed to evaluate mood and depressive symptoms over the preceding period. In this study, the 15-item short form (GDS-15) was used, with total scores ranging from 0 to 15. A score of 0–4 is generally considered within the normal range, 5–8 suggests mild depressive symptoms, 9–11 indicates moderate depression, and 12–15 indicates severe depression. Higher scores reflect greater severity of depressive symptoms. Participants with scores above the normal range were excluded to minimize the potential influence of depressive symptoms on cognitive performance in this study.

### 2.5. Blood Analysis

#### 2.5.1. Antioxidative Status Indicators

As a marker of lipid peroxidation, plasma 2-TBARS levels were measured using a commercial TBARS assay kit (no. 10009055, Cayman Chemical, Ann Arbor, MI, USA) according to the manufacturer’s instructions. Erythrocytic antioxidant enzyme activities, including superoxide dismutase (SOD), catalase (CAT), and glutathione peroxidase (GPx), were also measured using Cayman assay kits (Cayman Chemical, Ann Arbor, MI, USA) following the manufacturer’s protocols. The erythrocytic reduced form of glutathione (GSH) was measured as an indicator of the antioxidant capacity according to a method described by Griffith [32].

#### 2.5.2. Biochemical Analysis

Blood biochemical analyses included hematological parameters, lipid profiles, kidney function, liver function, and nutritional status. Complete blood cell analyses were performed using a hematology analyzer (XN-9000, Sysmex Europe, Norderstedt, Germany), and the remaining biochemical parameters were measured using an automated chemical analyzer (ADVIA^®^ Chemistry XPT System, Siemens Healthcare Diagnostics, Eschborn, Germany).

### 2.6. Dietary Intake Assessment

A registered dietitian conducted a 24 h dietary recall at baseline by interviewing participants and recording all foods and beverages consumed during the previous day. Participants were instructed to maintain their usual dietary habits throughout the intervention period. Dietary records were analyzed using nutrient analysis software (2020 version) provided by the Taiwan Dietitian Association (Taipei, Taiwan) to estimate energy, macronutrient, and micronutrient intake.

### 2.7. Compliance Monitoring

Participants were instructed to return any unused capsules at each follow-up visit (weeks 12 and 24). Adherence to the intervention was assessed by counting the remaining capsules at each visit.

### 2.8. Statistical Analysis

All data are presented as the mean ± standard deviation (SD) or *n* (%). Statistical analyses were performed using GraphPad Prism version 7.03 (GraphPad Software, San Diego, CA, USA). The Shapiro–Wilk test was used to assess normality. For normally distributed variables, within-group differences over time in the F and F+P groups were analyzed using paired *t*-tests, and between-group differences were analyzed using Welch’s *t*-test. For non-normally distributed variables, within-group differences over time were analyzed using the Wilcoxon signed-rank test, and between-group differences were analyzed using the Mann–Whitney U-test. Differences between categorical variables were assessed using the Chi-square test. A *p* value of < 0.05 was considered statistically significant.

## 3. Results

### 3.1. General Characteristics, Anthropometrics, and Blood Pressure (BP)

**Figure 1:**
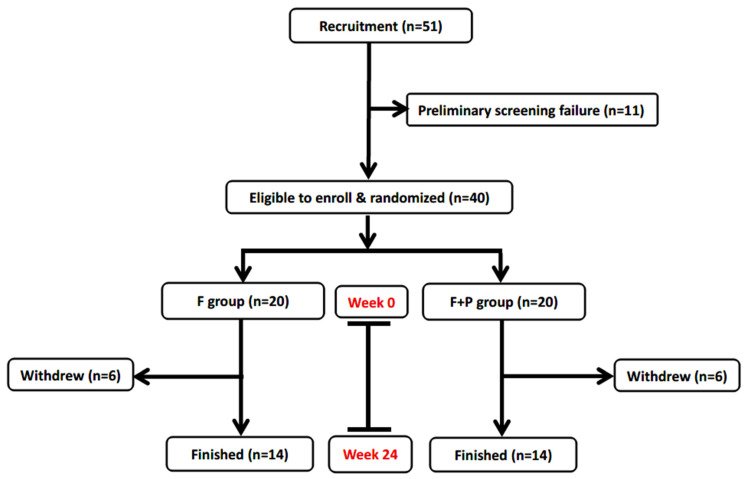
CONSORT flow diagram of participant screening and enrollment. In total, 51 participants were recruited. Participants with a CDR score ≤ 0.5 and an MMSE score of 26–30 were classified as having mild cognitive impairment (MCI). During the 24-week follow-up, in the F group, 2 participants withdrew before week 4, 2 before week 8, 1 before week 12, and 1 before week 24. In the F+P group, 2 withdrew before week 4, 3 before week 8, and 1 before week 16. All withdrawals were due to personal reasons unrelated to the intervention. Fourteen participants in each group completed the study, and analyses were conducted using data from these 28 participants.

Participant flow is shown in Figure 1. In total, 51 outpatients were referred for screening, of whom 11 did not meet the inclusion criteria. Forty participants with MCI provided written informed consent and were randomized to either the F group (*n* = 20) or the F+P group (*n* = 20). During the 24-week follow-up, 14 participants in each group completed the intervention and assessments. All withdrawals were due to personal reasons and were unrelated to the intervention.

As shown in Table 1, there were no significant differences between the two groups in sex distribution, age, or educational level. Regarding anthropometric outcomes, no significant changes were observed in the F group compared to the baseline. In the F+P group, body weight and BMI did not significantly change; however, body fat had significantly decreased, and muscle mass had significantly increased at the endpoint. There were no differences between the two groups in anthropometric measures at the baseline. At week 24, although body weight and BMI remained comparable between the groups, the F+P group had significantly lower body fat and significantly higher muscle mass than the F group. Regarding BP, no significant changes in SBP or DBP were observed in the F group after 24 weeks. In the F+P group, SBP was significantly reduced at week 24, whereas DBP showed no significant change. No significant between-group differences were observed at either the baseline or week 24.

### 3.2. Cognitive Function

As described above, MCI was defined as a CDR score of ≤0.5 and an MMSE score of 26–30. All participants in the present study had at least a primary school education, with a mean educational level of 14.1 ± 2.3 years. Based on the inclusion criterion, participants were within the cognitively normal to MCI range and had not reached the threshold for overt cognitive impairment.

As shown in Table 2, compared to the baseline, the F group showed a significant decrease in CDR scores and a significant increase in MMSE scores. In the F+P group, no significant changes in the CDR or MMSE scores were observed between the baseline and endpoint. No significant differences were found in the CDR or MMSE scores at either the baseline or week 24 between the two groups.

After 24 weeks of supplementation, CASI total scores showed no significant changes in either the F or F+P group (Table 2). In subdomain analyses, compared to week 0, the drawing score had significantly increased in the F group, while the short-term memory score had significantly increased in the F+P group after 24 weeks. It is noteworthy that although the drawing score in the F+P group at week 24 did not significantly differ from that at the baseline, it had already reached the maximum score. No significant between-group differences were observed in CASI total scores or in any subdomain scores at weeks 0 and 24.

Compared to week 0, GDS scores were significantly reduced in both the F and F+P groups. However, no significant differences in GDS scores were observed between the two groups at either week 0 or week 24.

### 3.3. Blood Biochemical Parameters

#### 3.3.1. Antioxidative Status

After 24 weeks, plasma TBARS concentrations had significantly increased in the F group, whereas the F+P group showed a slight, non-significant increase (Table 3). However, no difference was found between the F and F+P groups at weeks 0 or 24.

Regarding erythrocytic antioxidant enzyme activities, compared to week 0, CAT concentrations had significantly decreased in the F group after 24 weeks of supplementation, whereas no significant change was observed in the F+P group (Table 3). There was no significant difference in CAT activity between the two groups at the baseline; however, after 24 weeks of supplementation, CAT activity in the F+P group was significantly higher than that in the F group.

Compared to week 0, SOD activity had significantly increased at week 24 in the F group, whereas no significant change was observed in the F+P group between weeks 0 and 24. Between-group comparisons showed no significant differences in SOD activity at weeks 0 and 24.

GPx activity and GSH concentrations remained stable in both the F and F+P groups over the 24-week period, with no significant within-group changes from the baseline and no significant between-group differences observed at either week 0 or 24.

#### 3.3.2. Biochemical Indicators

Blood biochemical parameters are presented in Table 4 and were used to evaluate the safety of the supplementation. In the F group, no significant changes were observed from week 0 to week 24 in blood lipid profiles, renal and liver function markers, nutritional status indicators, or routine hematological parameters. In the F+P group, mean corpuscular hemoglobin (MCH) was significantly lower at week 24 compared to the baseline, although the values remained within the normal reference range. All other biochemical and hematological parameters showed no significant within-group changes over the 24-week period.

No significant between-group differences were observed in blood lipid profiles, kidney or liver function markers, or nutritional status indicators during the study (Table 4). Red blood cell counts significantly differed between the F and F+P groups at the baseline. After 24 weeks of supplementation, the mean corpuscular volume (MCV) and basophil counts significantly differed between the two groups; however, all values remained within normal reference ranges and were not considered clinically meaningful.

### 3.4. Dietary Intake 

Mean nutrient intake levels are presented in Table 5. Between-group comparisons showed that dietary fiber intake was significantly higher in the F+P group than in the F group. The F+P group also had significantly higher intake levels of vitamin A and magnesium. No other significant differences in nutrient intake were observed between the two groups.

### 3.5. Compliance

Mean compliance rates were 95% for both the fish oil and placebo capsules in the F group and 96% for both fish oil and pine bark extract capsules in the F+P group. No serious adverse events were reported in either group during the intervention period.

## 4. Discussion

Following enrollment, participants underwent comprehensive assessments, including physician diagnosis, cognitive evaluation, and dietary assessment. During the study, six participants from each group withdrew for reasons unrelated to the intervention, resulting in a retention rate of 70%. Overall, compliance was assessed based on returned capsules and was high in both groups (95–96%). To further support adherence, participants were provided with a study handbook containing instructions, follow-up schedules, and tracking sheets. Importantly, no serious adverse events were reported. In addition, no participants experienced notable gastrointestinal symptoms during supplementation with fish oil and pine bark extract, and all biochemical and hematological parameters remained within normal reference ranges (Table 4). Collectively, these findings indicate that the intervention was well tolerated.

In addition, according to the WHO Asian criteria, the BMI values of the F and F+P groups were within the normal to overweight range, whereas body fat percentages fell within the obesity range (Table 1) [33]. Elevated adiposity is associated with chronic inflammation and may contribute to neuroinflammation and neuronal injury [34]. In this context, after 24 weeks, no significant changes in body weight, BMI, body fat, or muscle mass were observed in the F group. Although previous studies have reported beneficial effects of n-3 PUFA supplementation on muscle mass and function [35], no such effects were observed in the present study, possibly due to differences in dosage or intervention duration. In contrast, combined supplementation with fish oil and pine bark extract reduced body fat and increased muscle mass (Table 1), suggesting a potential combined effect, possibly through improved redox balance. However, as physical activity was not objectively measured, the contribution of lifestyle factors cannot be excluded. Moreover, participants receiving antihypertensive medications were excluded due to the reported blood pressure-lowering effects of pine bark extract [36]. Although a significant reduction in SBP was observed in the F+P group, values remained within the normal range (Table 1), suggesting that the intervention did not lead to clinically adverse alterations in blood pressure and was well tolerated from a cardiovascular perspective.

On the other hand, participants in both groups met the criteria for MCI at baseline and week 24, with CASI total scores remaining within the normal range (80–100). After 24 weeks, the fish oil group showed significant improvements in CDR and MMSE scores, as well as in the drawing subdomain of the CASI (Table 2), suggesting potential benefits in visuospatial processing and executive function. Previous studies have also reported that n-3 PUFA supplementation may improve cognitive function in individuals with MCI [37,38]. However, variations in study design, intervention duration, and cognitive assessments may influence the observed outcomes. Collectively, these findings suggest that 24-week fish oil supplementation may help maintain cognitive function in individuals with MCI, with particular benefits in specific cognitive domains.

In contrast, after 24 weeks of combined supplementation with fish oil and pine bark extract, CDR and MMSE scores remained stable (Table 2). Although no significant change was observed in the CASI total score, the short-term memory subdomain showed a significant improvement, while the drawing subdomain had already reached a ceiling level. Previous studies have reported that pine bark extract or combined antioxidant supplementation may improve cognitive performance [39,40]. However, the effects appear to vary depending on study design and population characteristics. Taken together, these findings suggest that combined supplementation may help maintain global cognitive function while providing modest benefits in specific cognitive domains, such as short-term memory. Compared with fish oil alone, no additional improvement in global cognitive measures was observed. However, although statistically significant improvements were observed, the magnitude of the effects was modest. This may be due to the inclusion of participants at an early stage of cognitive decline, whose cognitive function was largely preserved at baseline. In such populations, intervention effects may be subtle and more likely reflected as a slowing of decline rather than marked improvement.

In addition, depression is common in individuals with MCI and may increase the risk of progression to dementia [41,42]. In this study, GDS was used to assess mood status, and individuals with elevated scores were excluded. After 24 weeks, GDS scores decreased significantly in both groups but remained within the normal range, with no between-group differences. These findings suggest that the observed cognitive changes were unlikely to be influenced by depressive symptoms.

Furthermore, in the F group, plasma TBARS levels increased, erythrocytic CAT activity decreased, and SOD activity increased after 24 weeks (Table 3). Fish oil, which is rich in highly unsaturated fatty acids, may increase oxidative burden and induce compensatory activation of endogenous antioxidant enzymes [17,18]. The increase in SOD activity likely reflects an adaptive response to enhanced ROS production rather than an overall improvement in oxidative status. Previous studies have similarly reported increased lipid peroxidation accompanied by compensatory antioxidant enzyme responses following PUFA supplementation [43,44]. The imbalance between increased SOD and reduced CAT activity may result in insufficient clearance of hydrogen peroxide, thereby contributing to lipid peroxidation.

In contrast, co-supplementation with pine bark extract appeared to provide additional antioxidant protection. Although TBARS levels slightly increased after 24 weeks, the increase was attenuated, and erythrocytic CAT activity was maintained in the F+P group, resulting in higher CAT activity than in the F group (Table 3). The pine bark extract used in this study is derived from Pinus pinaster Aiton (French maritime pine) and contains approximately 65–75% oligomeric proanthocyanidins (OPCs). In addition to OPCs, the extract includes other phenolic constituents such as catechin, caffeic acid, taxifolin, and ferulic acid. The compositional information was obtained from the manufacturer’s certificate of analysis [45], and no independent phytochemical analysis was performed in the present study. Although many previous clinical studies have used Pycnogenol^®^, a standardized extract derived from French Pinus pinaster, the extract used in this study is not identical and may differ in composition due to variations in extraction and standardization processes. Owing to its rich polyphenolic content, pine bark extract may contribute to improved redox balance by reducing lipid peroxidation and stabilizing antioxidant enzyme activity [46,47]. Taken together, the maintenance of CAT activity and the attenuated increase in TBARS in the F+P group suggest improved redox homeostasis. These findings indicate that while fish oil alone may induce compensatory antioxidant responses, the addition of pine bark extract may provide synergistic protection by stabilizing enzymatic defenses and reducing lipid peroxidation.

Moreover, the combined effects of n-3 PUFAs and pine bark extract may be better understood through a more integrated mechanistic framework beyond their conventional roles as direct antioxidants. Procyanidins from pine bark are extensively metabolized by gut microbiota into low-molecular-weight phenolic compounds, particularly phenyl-γ-valerolactones, which retain ortho-diphenolic structures and can undergo oxidation to form electrophilic quinones [48,49]. These electrophilic metabolites are capable of activating the Keap1-Nrf2 signaling pathway, thereby inducing endogenous antioxidant defense systems [50].

Similarly, n-3 PUFAs, due to their susceptibility to lipid peroxidation, can generate electrophilic lipid oxidation products such as α,β-unsaturated aldehydes, which have also been reported to activate Nrf2-mediated cytoprotective responses [51,52]. Therefore, rather than acting solely as direct radical scavengers, both pine bark-derived polyphenols and n-3 PUFAs may function as indirect antioxidants through electrophile-mediated activation of Nrf2 signaling pathways.

Importantly, these two classes of compounds differ in their physicochemical properties, which may influence their biological distribution and sites of action. Polyphenol-derived metabolites are generally hydrophilic and are likely to exert their effects within aqueous environments such as the cytosol and circulation, whereas n-3 PUFAs are lipophilic and primarily incorporated into cellular membranes, where lipid peroxidation processes occur. This distinction suggests a compartmentalized mode of action, whereby electrophile generation and subsequent Nrf2 activation may occur in different cellular or tissue environments. Such spatial complementarity may contribute to a more integrated activation of cellular defense systems, rather than a simple additive antioxidant effect.

Taken together, these mechanisms provide a more plausible biological rationale for the combined supplementation, highlighting the potential for complementary and coordinated regulation of oxidative stress-related pathways.

However, although combined supplementation improved antioxidant enzyme activity, this did not translate into significant cognitive benefits. Cognitive function is multifactorial, and changes in antioxidant status alone may not be sufficient to produce measurable effects within a limited period. Previous studies have reported inconsistent associations between antioxidant intake and cognitive outcomes [53,54]. Therefore, the relationship between antioxidant capacity and cognitive function may not be direct or immediate, and further studies are needed to clarify these associations.

Additionally, dietary intake was assessed using 24 h dietary recall to evaluate patterns during the intervention. Both groups showed a higher proportion of energy from fat and lower dietary fiber intake than recommended by WHO guidelines (Table 5) [55,56,57]. Although total energy and macronutrient intake did not differ significantly between groups, the F+P group had higher intakes of dietary fiber, vitamin A, and magnesium than the F group (Table 5). Previous studies have reported that high-fat dietary patterns, particularly those rich in saturated fats, are associated with an increased risk of cognitive decline and MCI [58,59,60]. Such diets may influence neuroinflammation, vascular function, and gut microbiota, which are key factors in brain aging and cognitive impairment [61,62]. On the other hand, emerging evidence suggests that higher dietary fiber intake is associated with a lower risk of cognitive impairment in older adults [63,64]. Insufficient fiber intake may contribute to cognitive decline, potentially through gut microbiota-mediated mechanisms [65,66]. Unfortunately, this study did not establish causal relationships between dietary patterns or nutrient intake and MCI, and the lack of a healthy control group should be acknowledged as a limitation. Nevertheless, the observed high fat intake and insufficient dietary fiber consumption among participants with MCI remain notable and may provide insights into their potential roles in MCI prevention.

Finally, this study has several strengths. A key strength is the use of a combined supplementation strategy, allowing the evaluation of potential complementary effects on cognitive and oxidative outcomes. The early stage following an MCI diagnosis represents a critical window for intervention [67]; therefore, a 24-week intervention was selected to capture early changes in cognition and oxidative status. Despite its relatively short duration, maintaining a 24-week trial in individuals with MCI is challenging. Notwithstanding the constraints of the COVID-19 pandemic, the study was successfully completed with repeated assessments of cognitive and oxidative biomarkers.

Several limitations should be acknowledged. First, the sample size was relatively small, and a placebo-only control group was not included, as all participants received fish oil. This limits the statistical power and generalizability of the findings and precludes a clear attribution of the observed effects solely to the intervention. Therefore, the results should be interpreted with caution. Second, dietary intake was assessed using a 24 h recall method, which may not reflect habitual long-term patterns. Third, circulating FA composition, inflammatory markers, and gut microbiota were not measured, limiting the interpretation of relationships between dietary fat intake, systemic FA status, inflammation, and cognitive outcomes. Fourth, the absence of a non-MCI or healthy control group, as well as a placebo control group, may limit the interpretation of the observed biochemical changes. As this is the first study to investigate combined supplementation with fish oil and pine bark extract in individuals with MCI, the findings should be considered preliminary. Fifth, oxidative stress-related markers such as NADPH oxidase, oxidized glutathione (GSSG), and GSH/GSSG ratio were not evaluated, partly due to limited sample availability, which limits a more comprehensive assessment of redox status and underlying mechanisms. Finally, although week 12 was planned as an interim assessment, complete blood and cognitive data were not available for all participants, partly due to COVID-19-related restrictions. No confirmed COVID-19 infections were reported among participants; however, the potential indirect impact of the pandemic cannot be excluded.

## 5. Conclusions

Compared with the F group, the F+P group showed reductions in body fat, increased muscle mass, and lower systolic blood pressure. Improvements were also observed in the short-term memory subdomain of the CASI. In addition, no significant increase in plasma TBARS was observed, and CAT activity was higher in the F+P group after 24 weeks. These findings suggest that combined supplementation with fish oil and pine bark extract may be associated with potential improvements in body composition, antioxidant status, and specific aspects of cognitive performance in middle-aged and older adults with MCI. However, due to the study design limitations, including the absence of a placebo-only control group and the relatively small sample size, the results should be interpreted as preliminary and hypothesis-generating. Further well-controlled studies are required to confirm these observations.

## Figures and Tables

**Table 1:** Characteristics, body composition and blood pressure in each group ^1^.

Characteristic	F (*n* = 14)		*p* Value ^2^	F+P (*n* = 14)		*p* Value ^2^	*p* Value ^3^	
Number of participants	14			14				
Gender								
Male, *n* (%)	2 (14%)			3 (21%)			0.622	
Female, *n* (%)	12 (86%)			11 (79%)				
Age (years)	63.7 ± 6.7			61.6 ± 5.1			0.365	
Education in years	11.9 ± 4.0			14.1 ± 2.3			0.088	
Height (cm)	157.5 ± 6.8			159.1 ± 7.6			0.539	
	Week 0	Week 24		Week 0	Week 24		Week 0	Week 24
Body weight (kg)	57.4 ± 7.6	57.6 ± 7.5	0.733	59.1 ± 9.8	59.0 ± 9.9	0.895	0.669	0.622
BMI (kg/m^2^)	23.9 ± 3.9	23.5 ± 3.2	0.460	23.2 ± 2.8	23.1 ± 2.7	0.724	0.598	0.742
Fat (%)	34.6 ± 4.2	35.2 ± 4.3	0.198	33.4 ± 3.1	31.9 ± 3.6	0.014	0.270	0.019
Muscle (%)	23.1 ± 2.2	22.7 ± 2.3	0.184	24.0 ± 2.5	24.7 ± 2.6	0.011	0.175	0.009
SBP (mmHg)	127.9 ± 13.9	124.2 ± 13.8	0.367	124.4 ± 18.4	116.9 ± 15.5	0.016	0.584	0.199
DBP (mmHg)	79.4 ± 7.3	78.5 ± 9.1	0.617	79.1 ± 9.4	77.6 ± 11.8	0.490	0.929	0.817

^1^ Data are expressed as the mean ± standard deviation or *n* (%). ^2^ *p* values represent comparisons between the baseline and endpoint in each group. ^3^ *p* values represent comparisons between the F and F+P groups at the baseline and endpoint. F, fish oil group; F+P, fish oil and pine bark extract group; BMI, body mass index; SBP, systolic blood pressure; DBP, diastolic blood pressure.

**Table 2:** Cognitive function assessment in each group ^1^.

	F group (*n* = 14)			F+P group (*n* = 14)			F vs. F+P	
	Week 0	Week 24	*p* Value ^2^	Week 0	Week 24	*p* Value ^2^	*p* Value ^3^	
							Week 0	Week 24
CDR	0.3 ± 0.3	0.1 ± 0.2	0.046	0.2 ± 0.3	0.2 ± 0.2	0.317	0.541	0.769
MMSE	28.5 ± 1.3	29.3 ± 0.7	0.046	28.4 ± 1.2	29.0 ± 1.0	0.233	0.884	0.511
CASI	93.2 ± 3.7	94.4 ± 4.2	0.133	94.1 ± 3.5	95.4 ± 3.1	0.181	0.538	0.511
LTM	9.6 ± 0.9	9.9 ± 0.5	0.317	9.9 ± 0.5	9.7 ± 0.7	0.564	0.541	0.769
STM	11.4 ± 0.8	11.6 ± 0.9	0.527	11.1 ± 0.7	11.7 ± 0.5	0.021	0.285	0.946
ATTEN	7.4 ± 0.9	7.6 ± 0.6	0.317	7.6 ± 0.8	7.6 ± 0.6	0.705	0.734	1.000
MENMA	9.4 ± 1.3	9.0 ± 1.3	0.389	8.9 ± 1.7	9.0 ± 1.5	0.671	0.376	0.804
ORIEN	17.3 ± 1.0	17.7 ± 0.7	0.180	17.4 ± 0.9	17.4 ± 0.9	1.000	0.769	0.541
ATRSTR	10.0 ± 1.2	10.1 ± 1.5	0.844	10.3 ± 1.3	10.9 ± 1.0	0.176	0.561	0.178
LANG	9.8 ± 0.4	10.0 ± 0.0	0.083	9.9 ± 0.4	9.9 ± 0.3	0.564	0.769	0.769
DRAW	9.7 ± 0.5	10.0 ± 0.0	0.046	9.9 ± 0.3	10.0 ± 0	0.336	0.352	0.769
ANML	8.5 ± 1.3	8.6 ± 2.0	0.794	9.1 ± 1.4	9.1 ± 1.5	1.000	0.210	0.603
GDS	3.5 ± 2.7	2.2 ± 2.3	0.007	4.4 ± 3.4	2.1 ± 2.7	0.020	0.423	0.981

^1^ Data are expressed as the mean ± standard deviation or *n* (%). ^2^ *p* values represent comparisons between the baseline and endpoint in each group. ^3^ *p* values represent comparisons between the F and F+P groups at the baseline and endpoint. F, fish oil group; F+P, fish oil and pine bark extract group; CDR, Clinical Dementia Rating Scale; MMSE, Mini-Mental State Examination; CASI, Cognitive Abilities Screening Instrument; LTM, long-term memory; STM, short-term memory; ATTEN, attention; MENMA, mental manipulation and concentration; ORIEN, orientation; ATRSTR, abstract thinking and judgment; LANG, language; DRAW, drawing; ANML, fluency; GDS, Geriatric Depression Scale.

**Table 3:** Antioxidative status in each group ^1^.

	F group (*n* = 14)			F+P group (*n* = 14)			F vs. F+P	
	Week 0	Week 24	*p* Value ^2^	Week 0	Week 24	*p* Value ^2^	*p* Value ^3^	
							Week 0	Week 24
TBARSs (μM)	6.6 ± 4.2	12.2 ± 15.4	0.032	5.2 ± 2.3	9.6 ± 11.3	0.063	0.276	0.615
CAT (nmol/min/mg)	562.6 ± 95.1	485.1 ± 75.9	0.004	544.9 ± 127.3	629.9 ± 161.7	0.078	0.680	0.007
SOD (U/mg)	1.5 ± 0.2	1.7 ± 0.4	0.016	1.5 ± 0.2	1.6 ± 0.6	0.950	0.849	0.085
GPx (nmol/min/mg)	14.8 ± 4.9	13.1 ± 4.9	0.186	13.1 ± 4.8	12.6 ± 5.8	0.658	0.367	0.818
GSH (nmol/mg)	4.4 ± 1.0	3.0 ± 2.3	0.109	4.3 ± 1.6	3.4 ± 2.3	0.309	0.821	0.646

^1^ Data are expressed as the mean ± standard deviation. ^2^ *p* values represent comparisons between the baseline and endpoint in each group. ^3^ *p* values represent comparisons between the F and F+P groups at the baseline and endpoint. F, fish oil group; F+P, fish oil and pine bark extract group; TBARSs, thiobarbituric acid reactive substances; CAT, catalase; SOD, superoxide dismutase; GPx, glutathione peroxidase; GSH, reduced glutathione.

**Table 4:** Blood biochemical parameters in each group ^1^.

	F group (*n* = 14)			F+P group (*n* = 14)			*p* Value ^3^	
	Week 0	Week 24	*p* Value ^2^	Week 0	Week 24	*p* Value ^2^	Week 0	Week 24
*Lipid profile*								
TGs (mg/dL)	115.7 ± 43.0	122.9 ± 42.5	0.606	120.9 ± 85.0	125.2 ± 68.9	0.778	0.667	0.734
TC (mg/dL)	212.4 ± 46.4	226.2 ± 45.9	0.228	194.6 ± 28.9	206.4 ± 47.2	0.431	0.233	0.271
HDL-C (mg/dL)	61.3 ± 18.6	61.4 ± 16.7	0.969	63.4 ± 14.5	58.4 ± 17.2	0.078	0.745	0.482
LDL-C (mg/dL)	128.7 ± 39.4	140.7 ± 41.3	0.246	111.9 ± 23.9	124.4 ± 48.1	0.391	0.187	0.343
TC/HDL-C	3.6 ± 0.8	3.9 ± 1.1	0.465	3.2 ± 0.8	3.8 ± 1.2	0.071	0.125	0.805
*Kidney function*								
Creatinine (mg/dL)	0.8 ± 0.1	0.8 ± 0.1	1.000	0.8 ± 0.2	0.8 ± 0.2	0.059	0.394	0.839
Uric acid (mg/dL)	5.7 ± 0.9	5.7 ± 0.9	0.767	5.1 ± 0.9	5.4 ± 1.2	0.348	0.101	0.502
*Liver function*								
AST (U/L)	24.1 ± 4.0	24.9 ± 7.4	0.679	28.1 ± 9.2	30.3 ± 28.0	0.414	0.164	0.910
ALT (U/L)	22.2 ± 5.4	24.6 ± 9.1	0.347	25.6 ± 8.7	24.8 ± 13.9	0.382	0.230	0.603
*Nutritional status*								
Albumin (g/dL)	4.5 ± 0.2	4.5 ± 0.2	0.542	4.5 ± 0.2	4.5 ± 0.3	0.439	0.570	0.669
*Hematology*								
WBCs (10^3^/μL)	5.9 ± 1.5	6.4 ± 2.2	0.239	5.9 ± 1.5	5.9 ± 1.8	0.835	0.920	0.535
RBCs (10^6^/μL)	4.4 ± 0.3	4.6 ± 0.3	0.086	4.8 ± 0.6	4.9 ± 0.5	0.645	0.035	0.083
Hemoglobin (g/dL)	13.7 ± 0.8	13.8 ± 0.8	0.548	13.8 ± 1.7	13.6 ± 1.8	0.581	0.856	0.757
Hematocrit (%)	40.4 ± 2.2	41.5 ± 2.4	0.072	41.0 ± 4.7	41.0 ± 4.5	1.000	0.662	0.735
*Hematology*								
MCV (fL)	91.0 ± 3.2	90.5 ± 1.9	0.404	85.4 ± 8.7	84.5 ± 8.6	0.090	0.044	0.008
MCH (pg)	30.9 ± 1.3	30.1 ± 1.4	0.027	28.7 ± 3.4	28.1 ± 3.3	0.036	0.077	0.085
MCHC (g/dL)	33.9 ± 0.7	33.3 ± 1.1	0.009	33.6 ± 1.0	33.2 ± 0.9	0.058	0.150	0.794
Platelets (10^3^/μL)	259.0 ± 69.5	267.4 ± 65.8	0.278	248.0 ± 54.8	242.7 ± 71.7	0.683	0.382	0.352
Neutrophils (%)	54.3 ± 10.2	56.2 ± 12.2	0.442	51.0 ± 8.9	49.9 ± 8.1	0.631	0.368	0.117
Lymphocytes (%)	37.3 ± 10.3	35.7 ± 11.2	0.950	39.0 ± 7.8	40.3 ± 7.2	0.512	0.285	0.209
Monocytes (%)	5.8 ± 0.8	5.7 ± 1.3	0.806	6.3 ± 1.2	6.0 ± 1.5	0.317	0.209	0.691
Eosinophils (%)	2.0 ± 1.0	1.8 ± 1.2	0.491	2.9 ± 1.8	3.1 ± 1.8	0.271	0.194	0.069
Basophils (%)	0.6 ± 0.3	0.5 ± 0.3	0.380	0.8 ± 0.3	0.8 ± 0.3	1.000	0.146	0.042
RDW-CV (%)	12.7 ± 0.7	12.7 ± 0.6	0.776	13.3 ± 1.3	13.4 ± 1.4	0.833	0.194	0.329

^1^ Data are expressed as the mean ± standard deviation or *n* (%). ^2^ *p* values represent comparisons between the baseline and endpoint in each group. ^3^ *p* values represent comparisons between the F and F+P groups at the baseline and endpoint. F, fish oil group; F+P, fish oil and pine bark extract group; TGs, triglycerides; TC, total cholesterol; HDL-C, high-density lipoprotein cholesterol; LDL-C, low-density lipoprotein cholesterol; ALT, alanine aminotransferase; AST, aspartate aminotransferase; MCV, mean corpuscular volume; MCH, mean corpuscular hemoglobin; MCHC, mean corpuscular hemoglobin concentration; RBCs, red blood cells; TIBC, total iron-binding capacity; WBCs, white blood cells; RDW-CV, red blood cell distribution width.

**Table 5:** Nutrient intake levels in each group ^1^.

Nutrient	F Group (*n* = 14)	F+P Group (*n* = 14)	*p* Value ^2^
Energy (kcal)	1583.4 ± 318.1	1681 ± 338.1	0.439
CHO (g)	181 ± 53.4	180.8 ± 52.2	0.994
CHO (kcal%)	45.9 ± 11.3	43.8 ± 9.8	0.679
Protein (g)	62.5 ± 14.7	61.0 ± 14.5	0.778
Protein (kcal%)	16.2 ± 4.7	14.7 ± 2.8	0.303
Fat (g)	68.5 ± 26.4	74.5 ± 17.3	0.479
Fat (kcal%)	38.2 ± 10.7	40.0 ± 5.5	0.583
MUFAs (g)	24.5 ± 12.3	25.6 ± 8.4	0.784
PUFAs (g)	18.3 ± 9.0	23.4 ± 8.7	0.139
n-6 FAs (g)	16.5 ± 8.2	21.2 ± 8.1	0.145
n-3 FAs (g)	1.5 ± 0.9	2.0 ± 1.1	0.221
SFAs (g)	20.9 ± 10.5	21.5 ± 6.1	0.847
Cholesterol (mg)	296.9 ± 184.4	217.4 ± 114.2	0.182
Fiber (g)	10.0 ± 4.3	15.8 ± 7.8	0.022
Vitamin A (μg RE)	898.3 ± 960.6	2312.6 ± 1948.5	0.039
Vitamin E (mg)	16.4 ± 7.2	21.6 ± 6.9	0.063
Vitamin C (mg)	89.8 ± 85.1	110.0 ± 90.7	0.748
Vitamin B1 (mg)	0.9 ± 0.4	1.1 ± 1.1	0.581
Vitamin B2 (mg)	1.1 ± 0.8	1.1 ± 0.5	0.871
Niacin (mg)	13.1 ± 6.3	14.1 ± 6.4	0.671
Vitamin B_6_ (mg)	1.2 ± 0.5	1.2 ± 0.6	0.981
Vitamin B_12_ (μg)	2.5 ± 2.3	2.1 ± 1.9	0.713
Folic acid (μg)	212 ± 96.9	270.8 ± 180.7	0.435
Sodium (mg)	1132.3 ± 549.3	1314.2 ± 1025.5	0.963
Potassium (mg)	1668.4 ± 722.7	2211.8 ± 910.4	0.092
Calcium (mg)	396.2 ± 348.7	622.4 ± 356.3	0.118
Magnesium (mg)	169.3 ± 71.5	249.9 ± 108.8	0.028
Phosphate (mg)	792.5 ± 345.1	905.9 ± 352.7	0.398
Iron (mg)	6.9 ± 3.2	9.3 ± 4.6	0.129
Zinc (mg)	7.2 ± 3.9	7.2 ± 2.6	0.646
Copper (mg)	0.1 ± 0.1	6.6 ± 24.4	0.381

^1^ Data are expressed as the mean ± standard deviation or *n* (%). ^2^ *p* values represent comparisons between the F and F+P groups. F, fish oil group; F+P, fish oil and pine bark extract group; CHO, carbohydrates; MUFAs, monounsaturated fatty acids; PUFAs, polyunsaturated fatty acids; n-6 FAs, n-6 fatty acids; n-3 FAs, n-3 fatty acids; SFAs, saturated fatty acids; RE, retinol equivalent.

## Data Availability

The data that support the findings of this study are available from the corresponding author upon reasonable request.

## Abbreviations

The following abbreviations are used in this manuscript:

ALT	alanine aminotransferase
ANML	fluency
AST	aspartate aminotransferase
ATRSTR	abstract thinking and judgment
ATTEN	attention
BMI	body mass index
CASI	Cognitive Abilities Screening Instrument
CAT	catalase
CDR	Clinical Dementia Rating
CHO	carbohydrate
DBP	diastolic blood pressure
DHA	docosahexaenoic acid
DRAW	drawing
EPA	eicosapentaenoic acid
GDS	Geriatric Depression Scale
GPx	glutathione peroxidase
GSH	reduced glutathione
HDL-C	high-density lipoprotein cholesterol
LANG	language
LDL-C	low-density lipoprotein cholesterol
LTM	long-term memory
MCH	mean corpuscular hemoglobin
MCHC	mean corpuscular hemoglobin concentration
MCI	mild cognitive impairment
MCV	mean corpuscular volume
MENMA	mental manipulation and concentration
MMSE	Mini-Mental State Examination
MUFA	monounsaturated fatty acid
n-3 FA	n-3 fatty acid
n-3 PUFA	n-3 polyunsaturated fatty acid
n-6 FA	n-6 fatty acid
OPC	oligomeric proanthocyanidin
ORIEN	orientation
RBC	red blood cell
RDW-CV	red blood cell distribution width
SBP	systolic blood pressure
SFA FA	saturated fatty acid fatty acid
SOD	superoxide dismutase
STM	short-term memory
TBARS	thiobarbituric acid reactive substance
TC	total cholesterol
TG	triglyceride
TIBC	total iron-binding capacity
WBC	white blood cell
WHO	World Health Organization
